# Influence of Sodium Acetate and Potassium Acetate on Alkaline Methanolysis for Biodiesel Synthesis

**DOI:** 10.1021/acsomega.5c04739

**Published:** 2025-09-17

**Authors:** Daniel A. R. de Campos, Érica B. de Sousa, Andreza D. M. Mendonça, Camila C. Lopes, Glauco F. Bauerfeldt, Matthieu Tubino, José G. Rocha

**Affiliations:** a Institute of Chemistry, Federal Rural University of Rio de Janeiro (UFRRJ), BR-465, Km 7, Seropédica , RJ 23.897-000, Brazil; b National Institute of Technology (INT), Av. Venezuela, 82, Rio de Janeiro , RJ 20081-312, Brazil; c Institute of Chemistry, University of Campinas (UNICAMP), P.O. Box 6154, Campinas , SP 13083-970, Brazil

## Abstract

Homogeneous alkaline catalysts are highly efficient for converting triglycerides to methyl esters under mild conditions. However, soap formation remains a significant limitation, reducing the yield and complicating separation. Shortening the reaction time is a key strategy to mitigate this issue. In this work, the effect of CH_3_CO_2_Na and CH_3_CO_2_K on the alkaline methanolysis of soybean oil catalyzed by sodium methoxide was investigated to reduce reaction time. A zero-order reaction kinetic model was employed. Observed rate constants (*k*_obs_) were determined from online monitoring the refractive index of the reaction mixture. Experiments were conducted at temperatures of 40.0, 50.0, and 60.0 °C and stirring speeds of 400 and 800 rpm. Both salts provided an increase in *k*_obs_ values compared to those in biodiesel synthesis in the absence of them. CH_3_CO_2_K proved to be the most effective additive by increasing *k*_obs_ values by up to 90% for methanolysis at 800 rpm and 40.0 °C, while CH_3_CO_2_Na increased values by up to 60% at 800 rpm and 40.0 °C. The conversion of triglycerides to methyl esters was determined by ^1^H nuclear magnetic resonance, and an increase in conversion of up to 2.9 and 2.6% was observed in the presence of CH_3_CO_2_K and CH_3_CO_2_Na, respectively, after 60 min of reaction. Inductively coupled plasma optical emission spectrometry (ICP-OES) analysis confirmed that Na and K levels in biodiesel were below 0.5 mg kg^–1^, well within international fuel quality limits. These findings highlight the potential of sodium and potassium acetates as effective additives for reducing the time of reaction and improving biodiesel synthesis.

## Graphical abstract

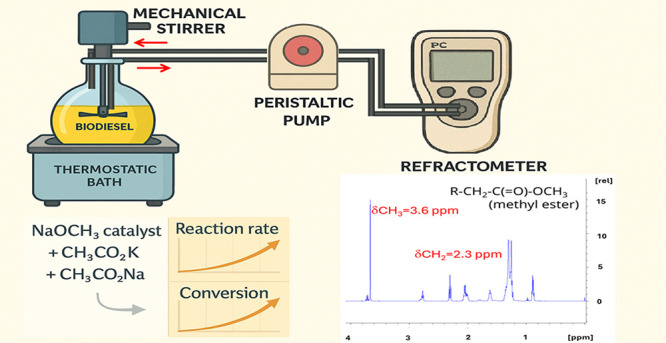

## 1 Introduction

Biodiesel is a renewable fuel mainly produced via transesterification of triglycerides with methanol (methanolysis), yielding glycerol as a byproduct.^1,2^ Homogeneous sodium-based catalysts are widely used in methanolysis. While NaOH stands out for its lower cost, NaOCH_3_ is the most attractive because it is purchased as a methanolic solution (eliminating the dissolution step) and produces less soap compared to NaOH or any other alkaline hydroxide.^3,4^ A major drawback of homogeneous alkali catalysis is saponification, caused by water or free fatty acids in the feedstock, which lowers biodiesel yield and generates emulsions that impair phase separation.^5^ The search for new catalysts has been encouraged to overcome such disadvantages as well as to obtain reusable catalysts and lower effluent generation.

Ion exchange polymeric resins,^6^ membranes,^7^ metallic complexes,^8^ inorganic oxides and salts,^9^ zeolites,^10^ enzymes,^11^ and ionic liquids,^12,13^ among others, have been recommended as alternative catalysts. However, no catalyst found in the literature was comparable to alkaline homogeneous catalysis in terms of rate of conversion (under moderate conditions), yield, and low cost to achieve broad and competitive practical application, despite the existence of production lines with alternative catalysts.^14^

Some authors related that the decrease in the time used in the synthesis of biodiesel with homogeneous alkali catalysts decreases the formation of soaps.^15^ This behavior can be justified by the fact that the saponification reaction is irreversible, occurring both in the esters of the raw material and in the esters produced in transesterification (i.e., biodiesel). Therefore, the search for favorable conditions to decrease the synthesis time is a way to minimize the inconveniences of alkaline catalysis due to the formation of soaps.

The methoxide ion is the main catalyst for methanolysis. It comes from the dissociation of alkaline methoxides (NaOCH_3_ or KOCH_3_) or from the reaction of alkaline hydroxides (NaOH or KOH) with methanol.^16^ In addition to the active species, the counterion also exerts an influence. Vicente et al. reported that sodium and potassium ions influence the conversion of triglycerides to biodiesel when alkaline hydroxides are used as catalysts,^17^ while Stanton et al. observed that these ions exert a kinetic effect in ester interconversion with alkaline *tert*-butoxides, a reaction that occurs through two successive transesterification steps.^18^ Because sodium and potassium ions influence both conversion and kinetics in biodiesel synthesis, adding salts containing these cations could accelerate methanolysis without requiring extra methoxide or hydroxide, thereby minimizing saponification.

Previous studies have progressively advanced our understanding of alkaline-catalyzed methanolysis. Tubino et al. developed an online refractometric method to continuously monitor biodiesel synthesis and compared pseudo-first-order and zero-order kinetic models.^3^ The zero-order model showed a better fit to the experimental data, which can be explained by the fact that methanolysis occurs predominantly at the methanol–oil interface, despite the solubility of the catalyst in methanol. Owing to the poor miscibility between methanol and oil, the reaction medium is not ideally homogeneous but rather a dispersion, where methanol droplets provide the surface on which the reaction takes place. Studies have confirmed this interfacial nature, demonstrating that droplet comminution by stirring increases the contact area and directly enhances the reaction rate.^19^ These findings provided a strong basis for adopting the zero-order model to describe the kinetics of biodiesel synthesis under alkaline catalysis.

Later, Tubino et al. performed a systematic comparison of NaOH, NaOCH_3_, KOH, and KOCH_3_ and demonstrated that potassium catalysts promote faster reactions than sodium analogues, and methoxides are more effective than hydroxides, findings attributed to ion-pair stability and differences in activation energy.^16^ More recently, Rocha et al. showed that dispersion of reactants consumes most of the overall synthesis time and is strongly influenced by the catalyst cation, with potassium species favoring more efficient dispersion than sodium.^20^ Together, these results highlight the critical role of cation effects and interfacial phenomena in biodiesel synthesis, supporting the current hypothesis that the addition of sodium and potassium salts can further modulate the kinetics of methanolysis by influencing the dispersion and ion-pair equilibria in the reaction medium.

In view of the reported effect of sodium and potassium ions present in homogeneous alkaline catalysts on the transesterification reactions and the need of alternatives to decrease the time required for the synthesis of biodiesel and, thus, decrease of the formation of soaps, the goal of the current study was to investigate the influence of CH_3_CO_2_Na and CH_3_CO_2_K on the kinetics of alkaline methanolysis for biodiesel production. The selection of these salts was based on their rapid solubilization and high solubility in methanol.

## 2 Materials and Methods

### 2.1 Materials

Biodiesel was synthesized using commercial soybean oil (acid value: 0.3 mg of KOH g^–1^). Methanol (99.5% w/w), potassium hydroxide (88% w/w), and sodium methoxide solution (30% w/w, in methanol) were obtained from Vetec, Brazil. Glycerol (99.5% w/w), sodium acetate (99.0% w/w), and potassium acetate (99.0% w/w) were obtained from Sigma-Aldrich, Brazil. All reagents were used as received. In this work, CH_3_CO_2_Na and CH_3_CO_2_K are called additives.

### 2.2 Biodiesel Synthesis

A 1 L round-bottom flask containing 300.0 g of soybean oil was added to a solution produced by dissolving 2.0 g of KOH in 60.0 g of methanol. The mixture was kept at 60 °C and vigorous magnetic stirring for 1 h (first transesterification). The mixture produced was transferred to a separatory funnel and left to stand for 30 min. Then, the biodiesel phase was reacted for 1 h at 60 °C with vigorous magnetic stirring, with a solution prepared by dissolving 0.50 g of KOH in 15.0 g of methanol (second transesterification). The mixture produced was placed in a separatory funnel and left to stand for 30 min. The biodiesel produced was washed with six portions of 250 mL of distilled water. This biodiesel was used to prepare a mixture designed to simulate the composition of the reaction mixture at the end of the reaction for the purpose of determining its refractive index.

### 2.3 Biodiesel Synthesis Monitoring

#### 2.3.1 Monitoring System

The progress of methanolysis was monitored by refractive index measurements, as the literature reports a strong correlation between refractive index and methyl ester content determined by GC-FID and ^1^H NMR.^21,22^

The system employed to monitor the alkaline methanolysis consisted of a round-bottomed flask (250 mL), a thermostatic bath (Tecnal, model TE-184, Brazil, ±0.1 °C), a peristaltic pump (Golander, model BT100F, USA), a mechanical mixer (Fisatom, model 713D, Brazil) with a 5 mm 4-blade propellers, and a refractometer (Mettler, model Refracto 30GS, Switzerland). A Teflon conical stopper with two holes was constructed to support the two silicone capillary tubes that conducted the reaction mixture in and out of the refractometer sample cell, allowing the reaction to be monitored in a continuous flow regime (Figure 1).

**Figure 1:**
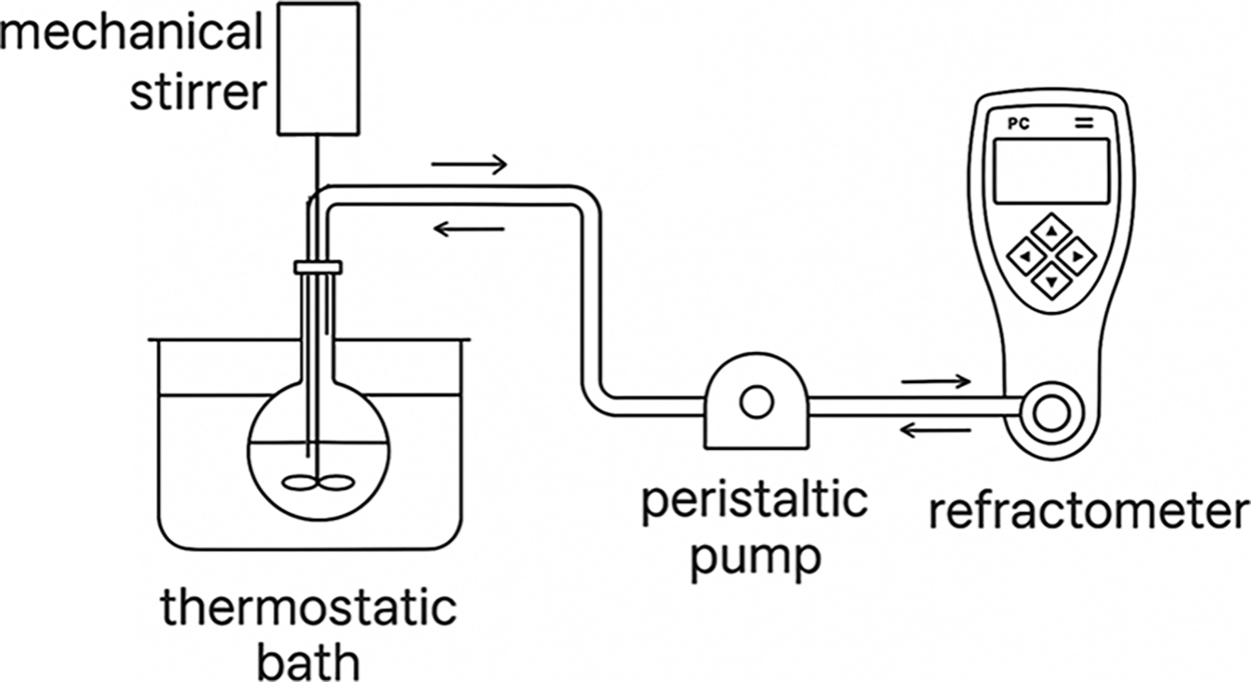
Continuous flow system used for reaction monitoring.

This system was similar to that used by Tubino et al.^3^ However, the system used here did not use the devices that separate the glycerol and bubble air from the reaction mixture before measuring the refractive index. The exclusion of the glycerol removal device is justified by the higher flow rate employed in the present system. While the original setup operated at 1 mL min^–1^, this work adopted a flow rate of 5 mL min^–1^. Under this condition, the residence time of the reaction mixture in the refractometer’s measurement cell is insufficient for phase separation and glycerol layer formation. Additionally, the direct connection between the reaction flask and the refractometer minimizes the risk of bubble formation along the flow path, eliminating the need for a bubble removal device.

#### 2.3.2 Reaction Procedure and Monitoring

A 250 mL round-bottomed flask containing 150.0 g of soybean oil was immersed in a thermostatic bath (at 40.0, 50.0, and 60.0 °C) and left under mechanical stirring (at 400 or 800 rpm). The oil was pumped to circulate in the monitoring system at a volumetric flow rate of 5 mL min^–1^. Methanolic solutions were prepared by mixing 2.9 mL of a sodium methoxide solution (equivalent to 16.1 mmol of NaOCH_3_) with 30.1 g of methanol and 10.2 mmol of additive. In each case, the flask containing the methanolic solution was immersed in the thermostatic bath. After the thermal equilibrium of the soybean oil and the methanolic solution was reached, the methanolic solution was quickly added to the round-bottomed flask containing oil, and the stopwatch was immediately started. Additionally, the monitoring of the biodiesel synthesis without additives was performed. Monitoring was performed in triplicate at the same experimental conditions.

#### 2.3.3 Reference Refractive Indices

In previous studies in which online monitoring of biodiesel synthesis was performed by measuring the refractive indices of the reaction mixture, it was observed that the initial variations in the refractive index were due to the homogenization of the reaction mixture, which precedes the methanolysis step.^3,20^ To monitor methanolysis, it is necessary to obtain the refractive indices of the reaction mixture at the beginning and end of the methanolysis.

Considering that methanol is not quite soluble in vegetable oil, it is important to emphasize that the term “homogenization” used in the present article must be understood as the idealized formation of a methanol/oil suspension with the minimal droplets size, i.e., with the maximal area of contact in the reaction conditions employed.

Two mixtures containing different proportions of soybean oil, biodiesel, methanol, and glycerol were prepared to simulate the composition of the reaction mixture at the beginning (0% conversion) and at the end (100% conversion) of the methanolysis of 150.0 g of soybean oil (Table 1). To calculate the quantities required for the preparation of these mixtures, it was considered that (i) soybean oil is composed 100% of triglycerides; (ii) the average molar masses of soybean triglycerides and of biodiesel are, respectively, 875 and 293 g mol^–1^;^23^ and (iii) the stoichiometric ratio of methanol to triglycerides is equal to 3:1. The mixtures were prepared in a 250 mL round-bottomed flask, which was inserted in the system used to monitor the biodiesel synthesis. After thermal equilibrium (at 40.0, 50.0, and 60.0 °C), stirring and pumping of the mixture in the system were activated and the monitoring of the refractive index began from this point until the end of the reaction of methanolysis.

**Table 1:** Mass, Grams, of the Reagents Used to Simulate the Beginning and End of the Methanolysis Reaction

mixture	conversion (%)	methanol	soybean oil	biodiesel	glycerol
1	0	30.10	150.0	0	0
2	100	13.66	0	150.45	15.75

The catalyst was not introduced in the preparation of mixtures 1 and 2 (Table 1) in order to avoid changes in the chemical composition of the system during the monitoring process.

Considering the relatively low amount of catalyst used in the synthesis, its contribution to the refractive index registered during the monitoring of the reaction can be neglected.

### 2.4 Evaluation of the Effect of Additives on Conversion to Methyl Esters

A round-bottom flask containing 150.0 g of soybean oil was heated to reaction temperatures (60, 50, and 40 °C) in a water bath. After thermal equilibrium, a methanolic solution prepared by mixing 30.1 g of methanol, 2.9 mL of NaOCH_3_ solution, and 10.2 mmol of additive (CH_3_CO_2_Na or CH_3_CO_2_K) was added to the oil into the round-bottom flask and kept under stirring (at 800 or 400 rpm), at reaction temperature, for 1 h. Then, the reaction mixture was transferred to a separatory funnel and left to stand for 30 min to separate the biodiesel and glycerol phases. The biodiesel was washed with six 100 mL portions of distilled water heated to 60 °C and dried in an oven (100 °C) for 3 h. This procedure was also performed without the catalyst (NaOCH_3_) to evaluate whether the additives have any isolated catalytic effect. All reactions were performed in duplicates.

The conversion of triglycerides to methyl esters was determined by ^1^H NMR. A Bruker Avance 500 spectrometer (United Kingdom) was used, with a magnetic field of 500 MHz, 45° radiofrequency pulse, 13 s pulse delay (acquisition time + relaxation time), scan width of 4.120 Hz, line width of 0.3 Hz, and 16 replicates. The biodiesel samples were dissolved in deuterated chloroform. The conversion was calculated by the equation

$$\mathrm{conversion}(\%)=\frac{2I_{3.6}}{3I_{2.3}}\times 100$$

where *I*_3.6_ is the area of the singlet of the methyl proton (at 3.6 ppm) directly linked to the carboxyl group of the methyl ester; *I*_2.3_ is the area of the signals of the alpha-carbonyl methylene proton (at 2.3 ppm); and the factors 2 and 3 are used to account for the number of H atoms present in the methyl and methylene groups, respectively.^24,25^

### 2.5 Determination of Sodium and Potassium Leaching

The determination of sodium and potassium potentially leached during biodiesel synthesis was carried out through inductively coupled plasma optical emission spectrometry (ICP-OES, Varian Vista-MPX) equipped with radial viewing, a V-Groove nebulizer, argon flow (15 L min^–1^), and a charge-coupled device (CCD) detector. Prior to analysis, the samples were diluted in kerosene. Quantification was performed using a multielement standard solution containing 100 mg kg^–1^ of Na^+^ and 100 mg kg^–1^ of K^+^. Calibration curves (0.5–5.0 mg kg^–1^) exhibited correlation coefficients above 0.995. The concentration limits adopted followed the specifications established by ABNT NBR 15553.^26^

## 3 Results and Discussion

### 3.1 Reference Refractive Indices for Methanolysis

To determine the initial and final refractive indices of system during the methanolysis, the refractive indices of mixtures 1 and 2 (Table 1) were monitored during their homogenization at 40.0, 50.0, and 60.0 °C, using the system shown in Figure 1. Figure 2A shows that the refractive indices gradually decreased during the homogenization of *mixture 1* due to the formation of the methanol/oil mixture and stabilized at values close to 1.4660, informing the values of the refraction indices that characterize the beginning of the methanolysis reaction. For *mixture 2*, the refractive indices stabilized at values close to 1.4500 (Figure 2B), corresponding to the end of methanolysis. In both cases (mixing and methanolysis), the reaction temperature had only a slight effect on the refractive index values after stabilization, possibly due to heat loss from the mixtures on the way to the refractometer.

**Figure 2:**
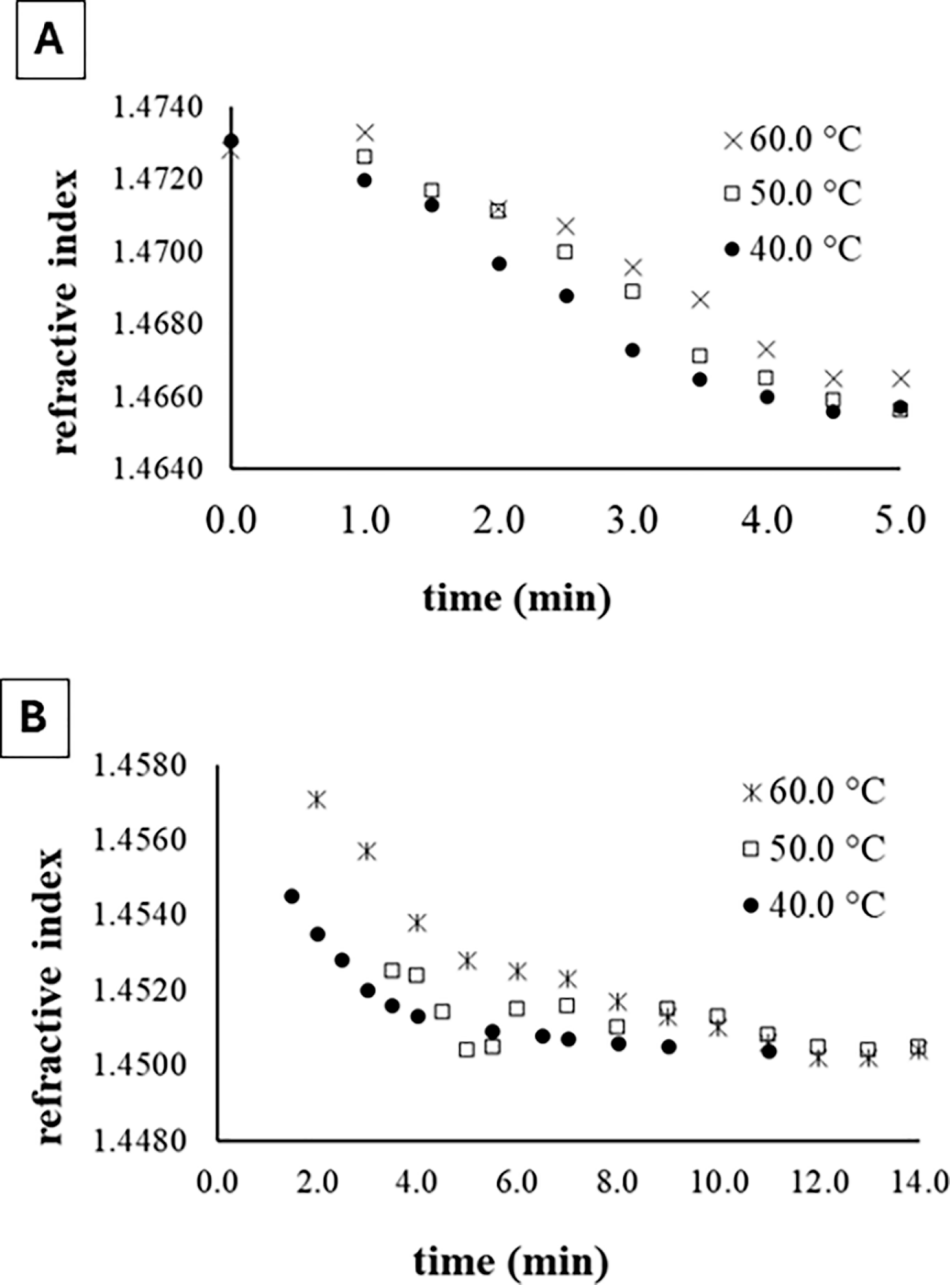
Monitoring the refractive indices during the homogenization of *mixtures 1* and *2*, with compositions similar to the beginning (A) and to the end (B) of the methanolysis, respectively.

### 3.2 Online Monitoring of Biodiesel Synthesis

Since the variations in the refractive indices obtained at the beginning of the monitoring are due to the homogenization of the reaction mixture,^3,16,20^ it was necessary to subtract the time spent for homogenization (*t*_H_) from the total time (*t*), introducing the variable *t′* (where *t′ = t – t*_H_), which represents the instant in which the refractive indices vary due to methanolysis. This variable is equal to zero (*t′* = 0) at the beginning of methanolysis (*n* ≈ 1.4660) and has a final value when methanolysis reaches equilibrium (*n* ≈ 1.4500). It is reasonable to consider that the transition from the homogenization step to the methanolysis step involves an intermediate stage, in which homogenization is being completed while methanolysis has already begun. Therefore, the curves obtained during monitoring were divided into three stages (Figure 3): homogenization; homogenization accompanied by methanolysis; and methanolysis. Thus, for the studies of the reaction kinetics, data obtained close to the end of monitoring were used, avoiding those affected by incomplete homogenization.

**Figure 3:**
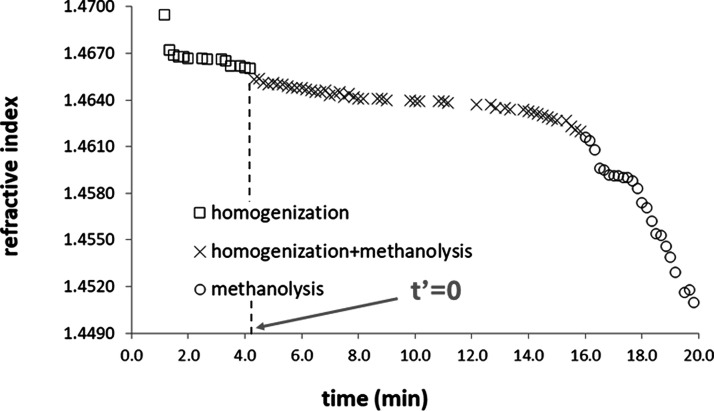
Monitoring of biodiesel synthesis at 40.0 °C, 400 rpm, and CH_3_CO_2_Na, highlighting the steps associated with homogenization, homogenization + methanolysis, and methanolysis.

### 3.3 Kinetic Study of Methanolysis

#### 3.3.1 Determination of Rate Constants

The methanolysis reaction was analyzed using the zero-order kinetics model (eq 1) previously reported in the literature,^3,16^ which adequately represents the interfacial character of this heterogeneous system.

$$\frac{\Delta n_{t’}}{\Delta n_{\mathrm{total}}}=-k_{\mathrm{obs}}t^{’}+1$$ 1

where *k*_obs_ is the observed rate constant, in ms^–1^; *t′* is the methanolysis time; Δ*nt′* is the variation of the refractive index from *t′* = 0 to any instant *t′* of methanolysis; and Δ*n*_total_ is the variation of the refractive index from the beginning to the end of methanolysis.

The linear correlation observed between the values of Δ*nt′*/Δ*n*_total_ versus *t′* (Figure 4) and the values of the correlation coefficients obtained (Figures 5 and 6) confirms that the methanolysis reaction, in the presence or absence of the additives, remains consistent with the zero-order kinetics previously demonstrated in the literature.^19,16^

**Figure 4:**
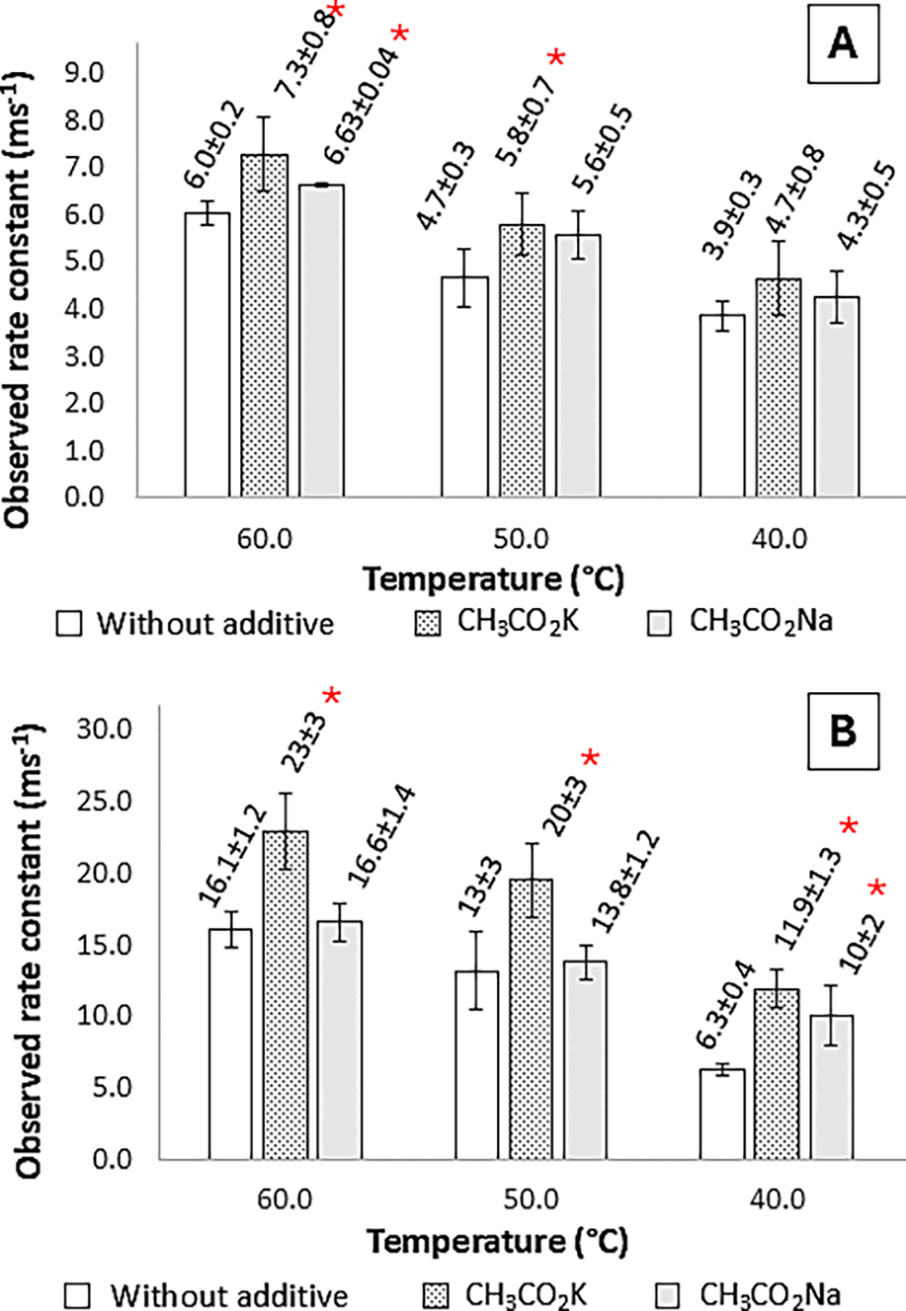
Values of observed methanolysis rate constants ± standard deviation, in ms^–1^, with and without additives at (A) 400 and (B) 800 rpm. *Significant increase (at α = 0.05) in the *k*_obs_ value compared to methanolysis without the additive, under the same reaction conditions.

**Figure 5:**
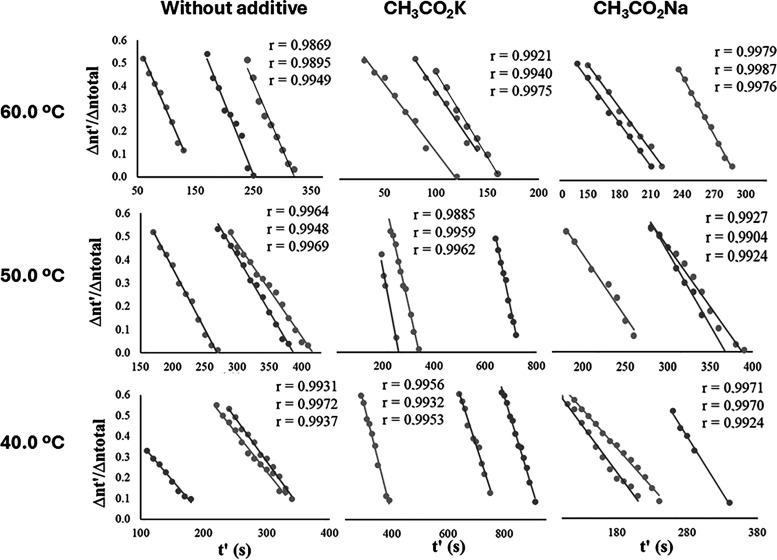
Plots of Δ*nt′*/Δ*n*_total_ versus *t′* to verify the validity of the zero-order kinetics model and determine *k*_obs_, in methanolysis at 400 rpm.

**Figure 6:**
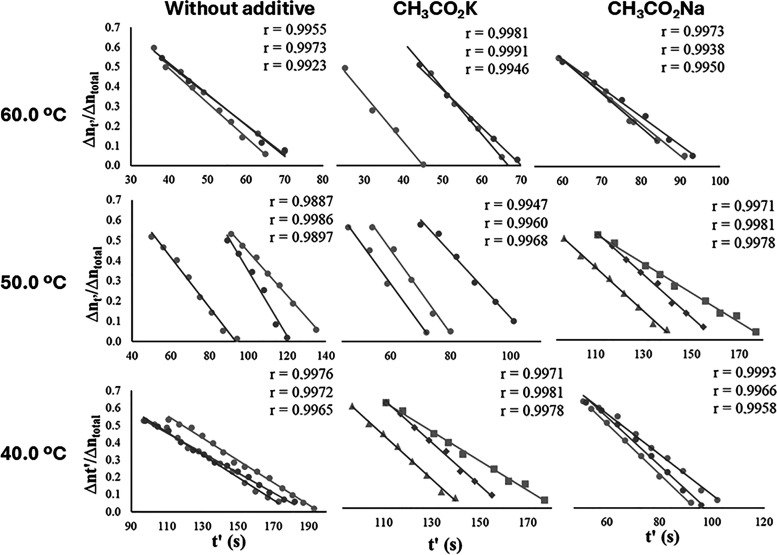
Plots of Δ*nt*′/Δ*n*_total_ versus *t′* to verify the validity of the zero-order kinetics model and determine *k*_obs_, of the reaction of methanolysis at 800 rpm.

The agitation speed was the variable that most influenced the values of the rate constant compared to temperature and the presence of additives. The *k*_obs_ values at 800 rpm were, on average, 2.7 times higher than at 400 rpm. This observation is consistent with a zero-order kinetics model, since a higher agitation speed promotes greater dispersion of methanol in the soybean oil phase, consequently producing a greater number of methanol droplets, which increases the contact area between the liquid phases.^27^ Since methanolysis occurs at the interface of the liquid phases,^3,28^ the higher agitation speed increases the reaction rate.

#### 3.3.2 Analysis of the Effect of Additives on Methanolysis

The average *k*_obs_ values in methanolysis at 400 rpm (Figure 4A) indicate that the additives promoted an increase in the reaction rate. Comparing the results with the *k*_obs_ values in methanolysis without the additives (at α = 0.05), it is possible to state that the *k*_obs_ value increased by 21, 24, and 10% using CH_3_CO_2_K at 50.0 °C, CH_3_CO_2_K at 60.0 °C, and CH_3_CO_2_Na at 60.0 °C, respectively. At 800 rpm (Figure 4B), the increases were statistically significant (α = 0.05) at 40.0 °C, corresponding to 90% with CH_3_CO_2_K and 60% with CH_3_CO_2_Na. At 50.0 and 60.0 °C, no important differences were observed in the *k*_obs_ values when using CH_3_CO_2_Na. The increase was statistically significant at 50.0 and 60.0 °C with CH_3_CO_2_K, corresponding to 54 and 43%, respectively. Therefore, from a kinetic point of view, CH_3_CO_2_K promoted a greater increase in the reaction rate compared to that of CH_3_CO_2_Na.

According to Tubino et al.,^16^ the methanolysis for biodiesel synthesis catalyzed by KOCH_3_ is faster than that catalyzed by NaOCH_3_, since the two catalysts in methanolic solution form the ionic pairs K^+–^OCH_3_ and Na^+–^OCH_3_, respectively. Gibbs free energy of the K^+‑^OCH_3_ ionic pair is higher than that of the Na^+‑^OCH_3_ pair, which leads to a lower activation energy for methanolysis catalyzed by KOCH_3_ compared to NaOCH_3_. This behavior explains the higher *k*_obs_ values achieved with CH_3_CO_2_K, compared to CH_3_CO_2_Na (Figure 4), in all reaction conditions employed, because the use of CH_3_CO_2_K leads to the formation of the K^+–^OCH_3_ ionic pair in methanolysis catalyzed by NaOCH_3_ (reaction 1), decreasing the activation energy of methanolysis in the presence of this catalyst.

CH_3_CO_2_K + Na^+–^OCH_3_ ⇌ CH_3_CO_2_Na + K^+–^OCH_3_ (reaction 1)

It was expected that the addition of CH_3_CO_2_Na would not promote a significant increase in the *k*_obs_ values in relation to methanolysis without additives, since the catalyst used was NaOCH_3_, a fact which did not occur for some reaction conditions (Figure 4). It is suggested that the increase in the ionic strength of the methanolic solution promoted by the addition of CH_3_CO_2_Na and CH_3_CO_2_K salts may offer some additional stabilization to the activated complex. This stabilization, potentially resulting from the development of partial charges within the activated complex, could reduce the activation energies. Therefore, both additives influence the *k*_obs_ values, albeit to different extents.

Since the reaction mixture is actually a heterogeneous system, due to the low miscibility between the reactants, with the formation of a biodiesel-rich phase and a glycerol-rich phase during the reaction, the distribution of methanol and catalyst between these phases exerts a kinetic effect on methanolysis.^29^ Therefore, since CH_3_CO_2_K and CH_3_CO_2_Na increase the ionic strength of the reaction medium, an increase in the amount of methanol and catalyst solubilized in the biodiesel phase may occur, potentially enhancing the catalytic activity.

### 3.4 Effect of Additives on Conversion of Triglycerides

The attempt to promote the synthesis of biodiesel without NaOCH_3_ (catalyst), even in the presence of CH_3_CO_2_Na or CH_3_CO_2_K, did not produce methyl esters, which was verified by the absence of the methyl proton of the −OCH_3_ group of the ester (at 3.6 ppm) (Figure 7). This fact clearly indicated that CH_3_CO_2_Na and CH_3_CO_2_K did not show catalytic activity. However, the additives, in the presence of the catalyst NaOCH_3_, promoted an increase in the conversion of triglycerides to methyl esters (Table 2). The increase in conversion after 60 min of reaction was more evident at lower temperature values and higher stirring speeds: 40.0 °C at 400 rpm, and both 40.0 and 50.0 °C at 800 rpm. Statistically significant increases (α = 0.05) in methyl ester conversions were observed in the presence of CH_3_CO_2_K at 40.0 °C and 400 rpm (1.3%), and with both additives at 40.0 °C (2.9% for CH_3_CO_2_K and 2.6% for CH_3_CO_2_Na) and at 50.0 °C (2.4% for CH_3_CO_2_K and 2.6% for CH_3_CO_2_Na), all at 800 rpm, when compared to reactions without additives.

**Figure 7:**
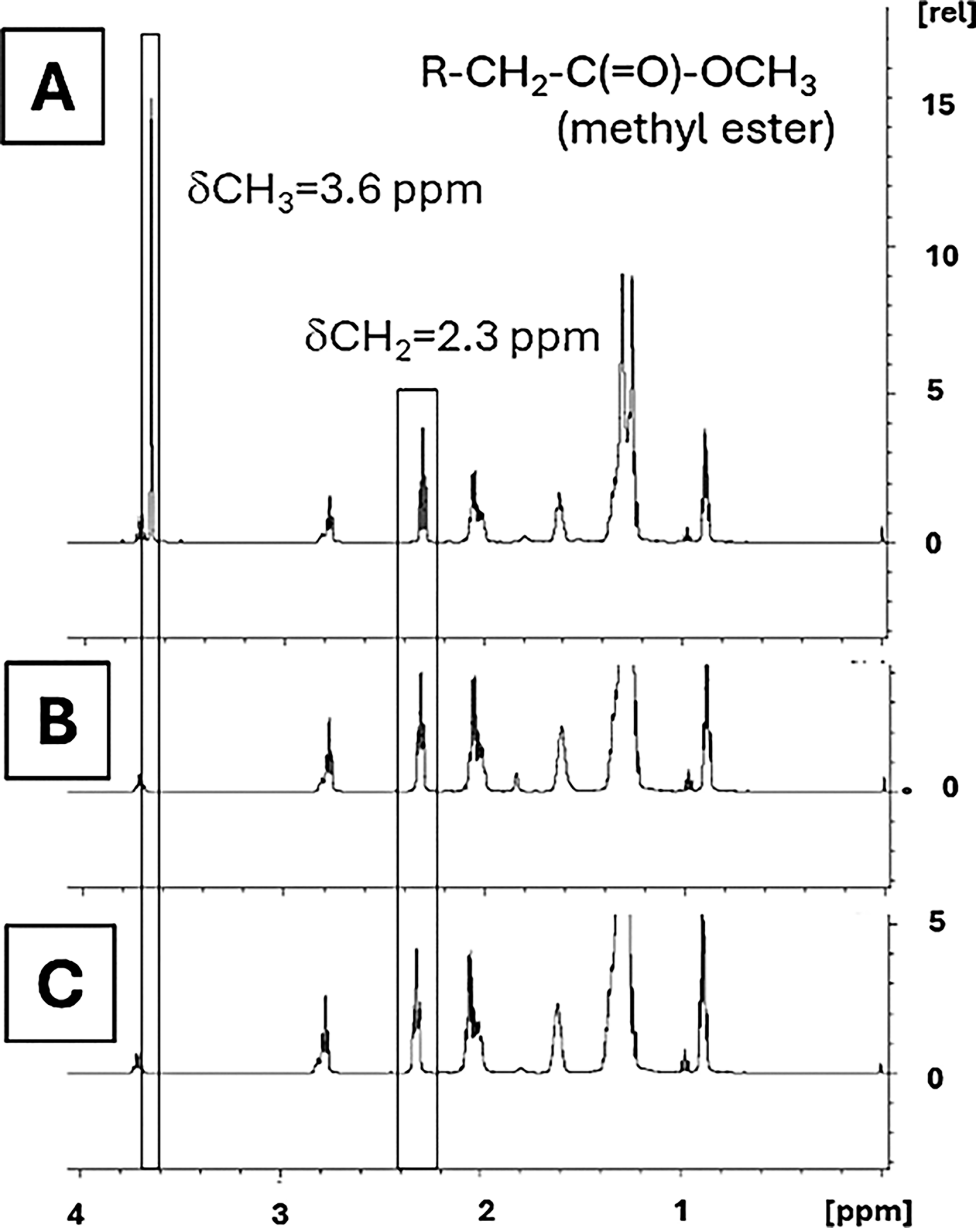
^1^H NMR spectra of biodiesel synthesis: (A) with NaOCH_3_ catalyst and without additives; (B) without NaOCH_3_ catalyst and with CH_3_CO_2_K; and (C) without NaOCH_3_ catalyst and with CH_3_CO_2_Na. R = fatty chain.

**Table 2:** Percentual Conversion of Triglycerides to Methyl Esters (±Standard Deviation) with and without Additives, after 60 min of Reaction

		temperature (°C)		
stirring speed (rpm)	additive	40.0	50.0	60.0
**400**	without	93.6 ± 0.3	95.12 ± 0.02	96.1 ± 0.4
	CH_3_CO_2_K	94.84 ± 0.10^a^	95.7 ± 0.5	96.54 ± 0.03
	CH_3_CO_2_Na	94.8 ± 0.5	95.48 ± 0.14	96.53 ± 0.11
**800**	without	92.47 ± 0.03	93.5 ± 0.4	96.22 ± 0.09
	CH_3_CO_2_K	95.4 ± 0.4^a^	95.9 ± 0.2^a^	96.62 ± 0.16
	CH_3_CO_2_Na	95.10 ± 0.04^a^	96.1 ± 0.2^a^	96.08 ± 0.13

a Significant increase (at α = 0.05) in the conversion value compared to methanolysis without the additive.

While kinetic studies showed that reaction rates were increased in the presence of additives, especially with CH_3_CO_2_K (Figure 4), conversion data indicated that under certain conditions, these enhanced rates led to higher final conversions of triglycerides to methyl esters.

The increased conversion of triglycerides into methyl esters provided by the studied additives opens prospects for biodiesel synthesis at lower temperatures than those traditionally employed (<60 °C) and with only one transesterification step, without compromising compliance with one of the quality specifications for its commercialization in Brazil and several European countries: the ester content (≥96.5% w/w).^30,31^ From a practical standpoint, this represents a significant advantage in terms of energy efficiency and process simplification, as it reduces the reaction time, catalyst consumption, and downstream processing. Based on the conversions obtained in syntheses performed in a single step, optimizing reaction parameters (such as reaction time, methanol-to-oil molar ratio, and the amounts of catalyst and additive) can contribute to ensuring that the resulting biodiesel complies with commercial specifications.

### 3.5 Sodium and Potassium Leaching in Biodiesel

The leaching of sodium and potassium ions into the biodiesel phase was evaluated under the conditions that provided the highest conversions (60 °C, 400 and 800 rpm), with and without the addition of sodium or potassium acetates. In all cases, Na and K concentrations were below 0.5 mg kg^–1^ of biodiesel, far lower than the maximum combined limits established by both European (EN 14214, Na + K ≤ 5 mg kg^–1^) and Brazilian (ANP 920/2023, ≤ 2.5 mg kg^–1^) specifications.^30,31^ These findings demonstrate that the use of sodium and potassium acetates does not compromise biodiesel quality with respect to the residual alkali metal content. On the contrary, the additives improved conversion and kinetics without introducing risks related to leaching, ensuring full compliance with international fuel quality standards.

## Conclusions

The addition of potassium and sodium acetates to the sodium methoxide-catalyzed methanolysis of soybean oil enhanced both the reaction rate and conversion to methyl esters. Kinetic data confirmed that the reaction follows a zero-order model and that stirring speed is the most influential variable. Potassium acetate, in particular, significantly increased the observed rate constant and final ester content, especially at lower temperatures and higher agitation. Importantly, Na and K contents in the biodiesel phase remained far below international limits, indicating that any leaching of these cations is negligible from a fuel quality perspective. These results demonstrate the potential of using such additives to produce biodiesel with a high ester content in a single reaction step.
